# Predicting Accuracy in Eyewitness Testimonies With Memory Retrieval Effort and Confidence

**DOI:** 10.3389/fpsyg.2019.00703

**Published:** 2019-03-29

**Authors:** Philip U. Gustafsson, Torun Lindholm, Fredrik U. Jönsson

**Affiliations:** Department of Psychology, Stockholm University, Stockholm, Sweden

**Keywords:** eyewitness accuracy, eyewitness testimony, confidence-accuracy relation, response latency, retrieval effort cues

## Abstract

Evaluating eyewitness testimonies has proven a difficult task. Recent research, however, suggests that incorrect memories are more effortful to retrieve than correct memories, and confidence in a memory is based on retrieval effort. We aimed to replicate and extend these findings, adding retrieval latency as a predictor of memory accuracy. Participants watched a film sequence with a staged crime and were interviewed about its content. We then analyzed retrieval effort cues in witness responses. Results showed that incorrect memories included more “effort cues” than correct memories. While correct responses were produced faster than incorrect responses, delays in responses proved a better predictor of accuracy than response latency. Furthermore, participants were more confident in correct than incorrect responses, and the effort cues partially mediated this confidence-accuracy relation. In sum, the results support previous findings of a relationship between memory accuracy and objectively verifiable cues to retrieval effort.

## Introduction

Eyewitness memories are often critical sources of information for investigating what happened during a criminal offense (Wells et al., 2006). Although playing a central role in criminal investigations and decision-making, eyewitness evidence has often been found to be unreliable, and constitutes a major contributing factor behind wrongful convictions (Garrett, 2011; Innocence project, 2018). Erroneous eyewitness reports are sometimes due to a witness’ deliberate lies about the target event (see DePaulo et al., 2003; Sporer and Schwandt, 2006; Vrij et al., 2017). Perhaps less obvious, and another major source of eyewitness error, is when a witness gives an honest report but remembers things incorrectly. While differentiating between sincere correct and incorrect memories may be critical to reaching valid judicial decisions, research has demonstrated that people have great difficulty in judging the accuracy of others’ memories (Lindholm, 2005, 2008a,b). Despite its importance to the judicial process, relatively little research has examined the extent to which erroneous eyewitness memories may differ from those that are accurate. The present study attempts to provide insight into potential differences between honestly reported correct and incorrect verbal eyewitness testimonies. We do this by replicating and extending the research of Lindholm et al. (2018), in which memory accuracy was found to be related to indicators of retrieval effort in witnesses’ responses.

### Means to Judge Memory Accuracy: Reality Monitoring and Cue-Utilization

While confidence in our own memories is not a perfect predictor of accuracy, research shows a consistent positive relationship between confidence judgments and memory accuracy (e.g., Robinson and Johnson, 1996; Odinot and Wolters, 2006; Wixted and Wells, 2017). *Reality monitoring* (Johnson and Raye, 1981) and *cue-utilization* (Koriat, 1997, 2006) are two major theories on how we make judgments of our own memories, that is, metamemory judgments. Both theories propose that we rely on indirect cues (i.e., heuristics) when assessing the veracity of our memory, rather than having a direct access to the memory’s strength (cf. Hart, 1965). Both theories have also inspired the development of methods for assessing the accuracy of others’ memories (e.g., Schooler et al., 1986; Sporer, 1997; Ackerman and Koriat, 2011). Reality monitoring theory (or “source monitoring”; Johnson et al., 1993) suggests that memories of real and imagined events differ in a set of attributes, and that people rely on these differences when determining the source of their memory. According to the theory, real memories include more contextual-, sensory-, and semantic information whereas imagined memories contain more references to cognitive operations. Reality monitoring can also be based on one’s prior knowledge and beliefs, such as judging a memory of a flying pig as imagined due to the knowledge that pigs cannot fly. Techniques using the reality monitoring framework have been developed to distinguish real from suggested memories (e.g., Schooler et al., 1986), and truth-tellers from liars (e.g., Sporer, 1997; Vrij, 2018). Since these techniques rely on patterns across several criteria in a testimony (e.g., sensory-, spatial-, time information, and clarity, etc.), they have primarily been used to determine the veracity of memories of entire events rather than of individual details from an event.

Similar to reality monitoring, the theory of cue-utilization (Koriat, 1997, 2006) suggests that people’s judgments of their own memories can be based on knowledge and beliefs about how memory works (information or theory-based), or on the experience derived during the retrieval process (experience-based). Experience-based judgments are mainly concerned with the memory processes *per se*, such as the ease with which the memory is retrieved, rather than, as within the reality monitoring framework, the content of the memory. While theory-based judgments within this framework are seen as derived from a deliberate application of one’s beliefs and theories about how memory works, experience-based judgments are derived on a more automatic basis from cues during the retrieval process. These cues give rise to a sense of experience from which the strength of the memory is estimated. Hence, a memory that comes to mind rapidly and easily would be experienced as a strong memory representation, and thus be judged as more accurate than one coming to mind more slowly.

Indeed, considerable evidence now attests to the notion that metamemory judgments, such as confidence, are strongly influenced by the ease and probability with which a to-be-remembered item is retrieved. For example, Kelley and Lindsay (1993) showed that manipulating how easy a memory is to retrieve affects how confident a person is that the memory is correct. In their study, participants were exposed to potential answers to general knowledge questions, which were either correct, incorrect but related, or incorrect and unrelated to the questions. When participants later took a test with the same questions, they were quicker to respond to, and more confident in answers they had been exposed to before, compared to non-exposed answers. This was true whether the answer was correct or incorrect, indicating the critical role of retrieval ease as a basis for their confidence judgments.

### Predicting Memory Accuracy

The vast majority of studies on eyewitness accuracy have focused on measuring and improving the accuracy of eyewitness identification, that is, witnesses’ ability to correctly recognize a perpetrator in a group of foils and suspects (see Wells et al., 2006). In these studies on recognition judgments, a witness’ subjective confidence in his/her memory is the most extensively researched factor (for reviews, see Brewer and Weber, 2008; Roediger et al., 2012; Roediger and DeSoto, 2014; Wixted et al., 2015; Wixted and Wells, 2017). Although it has been a matter of some debate over the years, the now prevailing view is that there is a consistent positive, albeit not perfect, relationship between confidence and recognition accuracy (Wixted et al., 2015; Wixted and Wells, 2017; see also Sporer et al., 1995; Juslin et al., 1996; Lindsay et al., 1998). Confidence has also been a prime interest in studies on verbal eyewitness recall, such as eyewitness testimony. While the strength of the relationship between confidence and accuracy in witness recall has varied somewhat throughout studies, the overall trend is consistent with, and mirrors the results of recognition studies; people are more confident in recalled memories that are correct, compared to incorrect (Robinson and Johnson, 1996; Robinson et al., 1997; Ibabe and Sporer, 2004; Odinot and Wolters, 2006; Odinot et al., 2009).

As explained previously, the cue-utilization view proposes that confidence judgments are not directly derived from the strength of memories but are based on internal (experience-based judgments) and external cues (information-based judgments), which are presumably related to a memory’s accuracy. However, if confidence is based on cues and not the strength of the memory itself, then the cues may constitute a more direct and valid relation to a memory’s accuracy than does confidence. Moreover, while confidence may be based on the indirect accuracy of cues, it seems plausible that the cues people rely on are not always those that are the most accurate predictors. Hence, if cues to a memory’s strength can be identified and measured, then such cues may provide a better estimate of accuracy than confidence judgments.

One cue that has been found to predict both accuracy and confidence is response latency, that is, the speed with which a memory is produced. As shown by Kelley and Lindsay (1993), people are more confident in quickly produced as compared to more slowly produced verbal responses. The same results were obtained in a study by Robinson et al. (1997), in which participants answered questions about details from a video of a staged theft. Higher confidence and shorter response latency for correct answers was found both for verbal recall as well as for recognition judgments. The relations between confidence, response latency and accuracy demonstrated in these studies in recall of episodic memories, are consistent with findings from a body of research on recognition of verbal information (Koriat and Ackerman, 2010; Ackerman and Koriat, 2011), semantic memory recall (Smith and Clark, 1993) as well as in eyewitness identification studies (e.g., Brewer et al., 2006; Weidemann and Kahana, 2016; for a review, see Brewer and Weber, 2008).

### Effort Cues as Accuracy Predictors

Given the evidence that memory accuracy is related to retrieval ease as measured by response latency, other cues of the ease with which a memory is retrieved should also predict accuracy. Lindholm et al. (2018) recently provided support for this notion. In two studies, participants were interviewed about their memory of a simulated crime event. In transcripts of these interviews, measures of effort were obtained by identifying a number of cues indicating retrieval difficulty. These effort cues included *delays* (pauses between or within statements), *hedges*, that is, commitment avoidance (e.g., “I think,” “maybe”), as well as *word fillers* (e.g., “well”) and *non-word fillers* (i.e., expressions without clear meaning, e.g., “uhm”). To control for the fact that a witness report typically includes both accurate and inaccurate information, effort and accuracy were estimated for witnesses’ statements about individual details from the target event, rather than the overall testimony (see also Ball and O’Callaghan, 2001). The results showed that effort cues were strongly related to honest witnesses’ memory accuracy, and that several of these cues contributed uniquely in predicting accuracy. While witness confidence was found to be positively related to accuracy, confidence did not contribute with any unique variance in predicting accuracy when the effort cues were included. Moreover, the effort cues fully mediated the relationship between confidence and accuracy, supporting the notion in cue-utilization theory that confidence is based on cues during memory retrieval, rather than a direct monitoring of memory strength (Koriat, 1997, 2006).

The finding of new, objectively verifiable cues that may be linked to eyewitness accuracy constitutes an important first step for developing methods to improve evaluations of eyewitness memory. However, before initiating attempts at methodological development, it is essential to further test the replicability of these initial findings. Moreover, while this first study examined temporal aspects of witnesses’ responses, this was not measured as the exact latency before a response as in previous studies, but rather in terms of a courser measure of delays before and during a response, unspecified with regard to length. It seems possible that the exact latency (a continuous measure) before initiation of a response is a more fine-tuned and better predictor of memory accuracy than a courser delay (discrete) measure, and that such a latency measure may even make other effort cues redundant. On the other hand, while response latency gives the exact timing before response initiation, pauses and hesitations during the response are not included in this measure. As memory retrieval is rarely instantaneous, but often unfolds as the memory is reported (Clark and Tree, 2002; Warren, 2012), delays during a response could also be critical cues to retrieval effort, and carry information about memories correctness. Thus, the role of response latency vs. other effort cues for determining eyewitness accuracy is an issue that warrants further clarification.

### The Current Study

The aim of the current study is to test the robustness of the Lindholm et al. (2018) findings, by a replication and extension of their research. Based on their results, it is hypothesized that retrieval effort cues (i.e., hedges, delays, and fillers) as well as confidence will predict memory accuracy. We further expect that confidence will not provide unique variance in predicting accuracy once the effort cues are accounted for. Extending the previous findings, the current study also measures the effort cue response latency and explores the contribution of this factor relative to the other effort cues in predicting accuracy. As the theoretical assumption from cue-utilization theory is that confidence is based on cues rather than derived from memory accuracy directly, we examined whether effort cues mediated the relationship between confidence and accuracy.

## Materials and Methods

### Participants

Twenty-two psychology students (15 female; *mean age* = 24.50 years, *SD* = 4.97) with normal or corrected-to-normal vision took part in the study in exchange for a movie voucher. Participants were informed that they were to see a simulated crime event on video, and that they would later be videotaped while being asked questions about the event. They all gave informed consent to participate.

### Materials and Procedure

The materials and procedures were identical to those carried out by Lindholm et al. (2018). Participants were tested individually in the lab, where they watched a 1-min film sequence involving a staged crime on a computer monitor. The film initially shows a man waiting at a bus stop. Shortly thereafter, a second man approaches the first man, attacks and stabs him in the gut, before leaving. After seeing the film, participants were interviewed about their memory of the event. The interviews included a free recall phase, immediately followed by a cued recall task with open questions (e.g., “how was the first man dressed?”). As the witness reported his/her memory, the interviewer wrote down the answers (e.g., “the offender had a green hat”) on a numbered sheet. Since the details reported by the witness were noted during an ongoing interview, it was not possible for the interviewer to catch every detail. Following the interview, the experimenter read out the details the witness had reported, and after each one, the witness wrote down his/her confidence in the accuracy of the statement, ranging from 0 to 100%, on a sheet with numbers corresponding to that of the experimenter. We asked for confidence after the interview had finished to allow witnesses to make a focused memory search without being interrupted repeatedly. This also allowed us to better mimic a free-recall situation similar to that typical of eyewitness testimony. As we were interested specifically in cues to accuracy in memories of individual details, rather than in overall accuracy, witnesses did not provide overall confidence estimates, neither in free nor cued recall.

The videotaped interviews were then transcribed verbatim (including fillers like “uhm,” “uh,” and self-talk). Based on the information in the crime video, we first cataloged all scorable and objectively verifiable details. An example of such verifiable detail is “He wore sneakers” whereas “He was cold” is a detail that could not be verified objectively. Based on this catalog, participants’ responses were then coded for accuracy by two independent raters (interrater reliability *r* = 0.75). Responses to the cued recall questions were then inspected, and two new independent coders selected all statements that provided either accurate or inaccurate information about a verifiable detail in response to a question (interrater reliability *r* = 0.95). Statements including partly correct and partly incorrect information (e.g., “he was wearing a white [incorrect] jacket [correct]”) were excluded.

Given that questions in the cued recall phase sometimes asked for a detail the participant had mentioned during free recall, we focused on responses during cued recall to avoid associating the same confidence score to two different reports of the same information. This yielded a total of 790 correct answers and 253 incorrect statements. Of these, confidence was obtained for 275 correct and 103 incorrect statements. To make our results section less convoluted, we focus our analyses only on statements for which confidence ratings were made. Next, two new blind coders coded the frequency of verbal and paraverbal expressions of effort in in each statement. Both coders coded the entire set of statements, and inconsistencies were resolved by a third coder. For these effort codings, we calculated the agreement between coders both with Cohen’s kappa (κ), as well as the percentage of exact overlap, that is, the degree to which codings of the cues by one coder corresponded with regard to both cue type and exact cue position in each testimony coded by the other coder. Using the operationalizations by Lindholm et al. (2018) (see Table 1), the following effort cues were coded: (1) *Non-word Fillers* – interjections and sounds like “hm,” “uh,” etc. (interrater reliability Cohen’s κ = 0.97, exact overlap = 91%); (2) *Word Fillers* – e.g., “meaningless” words like “you know,” “well,” etc. This category also included self-talk such as “Let’s see...” (interrater reliability Cohen’s κ = 0.83, exact overlap = 65%); (3) *Hedges* – word forms that reduce the force of an assertion, allow for exceptions, or avoid commitment, such as “I think” and “maybe” (interrater reliability Cohen’s κ = 0.87, exact overlap = 62%). We also measured *Delays* – a pause longer than 2 s before or during a response. Finally, we measured a fifth effort cue, *Response latency* (see Table 1). Both response latency and delays were measured using the video editing software iMovie (version 10.1.10, Apple Inc., 2018). The interviews of the participants were loaded into the program, and elapsed time was obtained by computing the temporal distance of silences between utterances as indicated by sound wave intensity. Hence for these cues, interrater reliability was not measured.

**Table 1:** Operationalizations of the effort cues in the witnesses’ responses.

Delays	A pause longer than 2 s before or during a response.
Non-word fillers	Interjections and sounds like “uh,” “hm,” sighs, “pff,” etc.
Word fillers	“Meaningless” words like “you know,” “well,” “so,” “so to speak,” etc. Also includes self-talk “Let’s see...,” “What was it?”
Hedges	Word forms that reduce the force of an assertion, allow for exceptions, or avoid commitment, such as “I think,” “maybe,” “sort of,” “could,” “something like that.”
Response latency	Elapsed time (in seconds) between the end of the interviewer’s question and the initiation of the witness’ response, or the time between the end of one statement from the witness and the start of a new witness statement.

## Results

### Predicting Accuracy With Effort Cues and Confidence

**FIGURE 1:**
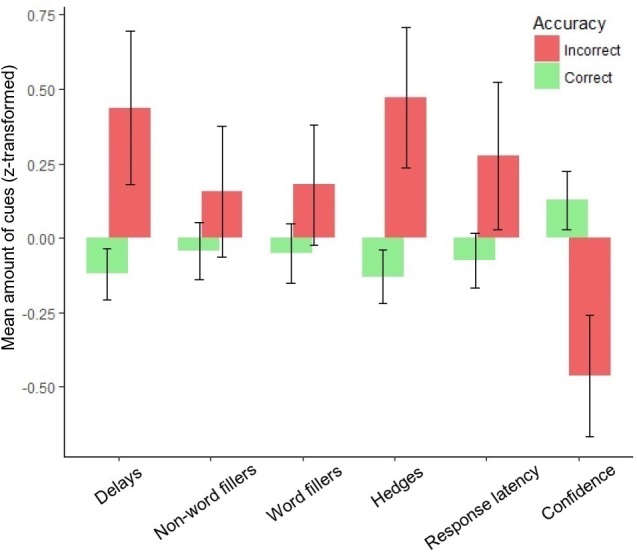
Mean amount of retrieval effort cues and confidence (*z-*transformed) in correct and incorrect memories. Error bars represent 95% confidence intervals.

Mean amounts of effort cues and confidence (*z-*transformed) in accurate and inaccurate statements for each variable are presented in Figure 1.

As the design used repeated measures (all participants provided both correct and incorrect responses), in combination with a varying number of responses produced by different participants, data were therefore organized as a multilevel data set with individual responses nested within participants (Wright and London, 2009). The calculations were computed with R (R Core Team, 2018), using the *lme4* package (Bates et al., 2015).

Our analyses largely followed the procedure outlined in Field (2009) and Mansour et al. (2017). Hence, we first ran a set of regressions to examine which individual variables predicted accuracy. Thus, a baseline, intercept-only model predicting accuracy (Model 1) was compared with models including each effort cue and confidence separately (Models 2–7). Table 2 illustrates the model parameter estimates and fit indices. In this table, effect sizes are given as Akaike Weights. The Akaike Weights varies between 0 and 1 and estimate the probability that the chosen model is the best-fitting model, relative to the other model(s) (Burnham and Anderson, 2004; Wagenmakers and Farrell, 2004). Hence, larger values indicate better fit. The results showed that model fit was significantly improved compared to the baseline model when adding *Delays*, χ^2^(1) = 22.37, *p* < 0.001, w_i_(AIC) = 0.99; *Word Fillers*,χ^2^(1) = 3.88, *p* = 0.048, w_i_(AIC) = 0.72; *Hedges*, χ^2^(1) = 26.30, *p* < 0.001, w_i_(AIC) = 0.99; and *Confidence*, χ^2^(1) = 27.95, *p* < 0.001, w_i_(AIC) = 0.99, but not by adding *Non-word Fillers*,χ^2^(1) = 2.94, *p* = 0.088, w_i_(AIC) = 0.61. In addition, *Response latency*, χ^2^(1) = 8.93, *p* = 0.003, w_i_(AIC) = 0.97, improved fit compared to the baseline model.

**Table 2:** Parameter estimates for predictors in models of accuracy (478 observations).

Predictor	Model 1	Model 2	Model 3	Model 4	Model 5	Model 6	Model 7	Model 8^†^	Model 9
**Fixed effects**								
Intercept	1.29 (0.11)^∗∗∗^	1.61 (0.14)^∗∗∗^	1.39 (0.12)^∗∗∗^	1.39 (0.13)^∗∗∗^	1.72 (0.16)^∗∗∗^	1.56 (0.15)^∗∗∗^	−0.45 (0.36)	0.70 (0.47)	0.76 (0.49)
Delays		−0.97 (0.21)^∗∗∗^						−0.69 (0.23)^∗∗^	–
Word fillers			−0.33 (0.16)^∗^					−0.11 (0.19)	−0.18 (0.18)
Non-word fillers				−0.23 (0.13)				–	–
Hedges					−0.64 (0.13)^∗∗∗^			−0.35 (0.15)^∗^	−0.42 (0.15)^∗∗^
Response latency						−0.21 (0.07)^∗∗^		–	−0.14 (0.07)
Confidence							0.02 (<0.01)^∗∗∗^	0.01 (<0.01)^∗∗^	0.01 (<0.01)^∗∗^
**Random parameterss**								
Level 2 intercept variance (participant)	<0.001 (<0.001)	<0.001 (<0.001)	<0.001 (<0.001)	<0.001 (<0.001)	0.04 (0.19)	<0.001 (<0.001)	0.13 (0.36)	0.11 (0.33)	0.10 (0.31)
**Model fit**								
Model df	2	3	3	3	3	3	3	6	6
Test change in df		1^a^	1^a^	1^a^	1^a^	1^a^	1^a^	3^b^	3^b^
AIC	502.20	481.83	500.32	501.26	477.90	495.27	476.25	463.38	468.76
BIC	510.54	494.34	512.83	513.77	490.41	507.78	488.76	488.39	493.78
Akaike weight	3.74 × 10^−9^	9.35 × 10^−5^	9.35 × 10^−9^	5.61 × 10^−9^	6.54 × 10^−4^	9.35 × 10^−8^	0.001	0.93	0.06
−2 log likelihood	−249.10	−237.91	−247.16	−247.63	−235.95	−244.63	−235.13	−225.69	−228.38

*Standard errors for fixed effects and standard deviations for random effects are given in parentheses. df, degrees of freedom; AIC, Akaike Information Criterion; BIC, Bayesian Information Criterion. ^†^Best-fitting model. Superscripts indicate df for the comparison between the current model and ^a^ Model 1 (baseline model), ^b^ Models 2–7. Asterisks indicate unique predictors within the model, ^∗^ p < 0.05, ^∗∗^ p < 0.01, ^∗∗∗^ p < 0.001.*

We next examined whether a model including all the significant variables from the first set of regressions improved fit relative to each of the separate models with significant predictors. Because delays and response latency were both significant, but partly based on the same data (a 2-s pause before the beginning of a statement would be coded both as latency and as a delay), we first needed to determine which of the two would be optimal in a model including all significant variables (we also checked for multicollinearity between all cues, and only response latency and delays were at risk, see Supplementary Table 1). Hence, we ran a model including Hedges, Delays, Word Fillers and Confidence (Model 8), and a model in which Delays were swapped for Response latency (Model 9), and compared the two models’ fit to data (see Table 2 for parameter estimates and fit indices). To assess which model had the best fit, we compared Akaike Weights for each model. The results showed that Model 8 including *Delays* [w_*i*_(AIC) = 0.93] had a better fit, compared to Model 9 with *Response latency* [w_*i*_(AIC) = 0.06, see Table 2]. In the subsequent analysis, therefore, we used the model with Hedges, Delays, Word Fillers, and Confidence and compared it to the models with each significant predictor.

Results showed that our model with multiple predictors significantly improved fit compared to the models with only Hedges, χ^2^(3) = 20.52, *p* < 0.001, w_*i*_(AIC) = 0.99; Delays, χ^2^(3) = 24.45, *p* < 0.001, w_i_(AIC) = 0.99; Word Fillers, χ^2^(3) = 42.95, *p* < 0.001, w_*i*_(AIC) = 0.99; and Confidence, χ^2^(3) = 18.88, *p* < 0.001, w_*i*_(AIC) = 0.99. The best-fitting model thus contained Hedges, Delays, Word Fillers, and Confidence. In this model, *Delays* (*z* = 2.97, *p* = 0.003) and *Hedges* (*z* = 2.23, *p* = 0.026) decreased as accuracy increased, proving unique predictors of memory accuracy, whereas *Word Fillers* (*z* = 0.60, *p* = 0.548) did not (see Table 3). Moreover, and contrary to expectations, *Confidence* contributed uniquely in explaining memory accuracy when controlling for the other predictors (*z* = 2.72, *p* = 0.007), increasing with increased accuracy.

**Table 3:** Multilevel logistic regression analysis predicting response accuracy from effort cues and confidence (*z*-transformed).

				*95% CI for OR*	
*Predictor*	*B (SE)*	*Z*	*OR*	*LL*	*UL*
**Delays**	**−0.35 (0.12)**	**2.97^∗∗^**	**0.70**	**0.56**	**0.89**
Word fillers	**−**0.07 (0.11)	0.60	0.93	0.75	1.17
**Hedges**	**−0.29 (0.13)**	**2.23^∗^**	**0.75**	**0.58**	**0.97**
**Confidence**	**0.38 (0.14)**	**2.72^∗∗^**	**1.47**	**1.11**	**1.94**
*Model fit*^1^	*AIC = 463.4, BIC = 488.4*,χ^2^(*4) = 46.82*^∗∗∗^	

*Parameters whose CI of B do not include zero (i.e., whose CI of OR do not include 1) are boldfaced. B, logistic coefficients; SE, standard error of the logistic coefficients’ estimation; z, z-value of coefficient; OR, odds ratios [Exp(B)]; CI, confidence intervals; LL, lower limit; UL, upper limit. ^∗^p < 0.05, ^∗∗^p < 0.001, ^∗∗∗^p < 0.001. ^1^Model fit compared to a baseline, intercept only model.*

### Effort Cues as a Basis for Confidence

**FIGURE 2:**
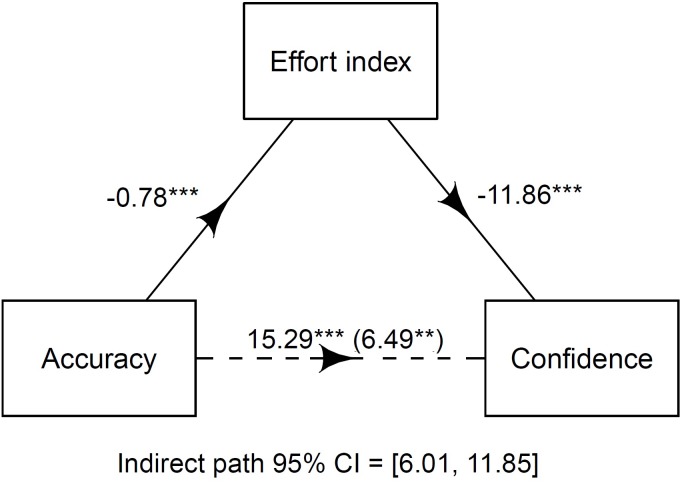
Effort index as a mediator of the relationship between accuracy and confidence. Values represent unstandardized parameter estimates for each path. Along the path from accuracy to confidence the numbers in parentheses represent the coefficients when the effort index was entered into the analyses. Dashed line indicates that the direct path is significantly mediated by the indirect path. ^∗∗^*p* < 0.01, ^∗∗∗^*p* < 0.001.

In the final analysis, we examined the role of effort cues as mediators of the relationship between accuracy and confidence. For this analysis, we created an effort index by summarizing hedges and delays, the two effort cues that uniquely predicted accuracy. The mediational analysis was run using the *mediation* (Tingley et al., 2014) package. Results showed that the effort cues partially mediated 57.3% of the relation between accuracy and confidence (see Figure 2).

The datasets analyzed for this study, and the code for the analyses, have been deposited in the Open Science Framework. Link to datasets: https://osf.io/uthbz/?view_only=1284f5b56d6d4af58679c74d913351fc. Link to code for analyses: https://osf.io/8kjnv/?view_only=baadf99fa8f7446e989f04d9a5e344bf.

## Discussion

The aim of this study was to further explore previously demonstrated relations between eyewitness accuracy and cues to retrieval effort (Lindholm et al., 2018). Our results largely replicate previous results, providing additional support for the use of effort cues in estimating eyewitness accuracy. Looking at the relationship between accuracy, effort cues and confidence, we found that effort cues partially mediated the relationship between confidence and accuracy (Figure 2). This study also measured the effort cue response latency, and found, in line with previous studies (Brewer et al., 2006; Koriat and Ackerman, 2010; Ackerman and Koriat, 2011; Weidemann and Kahana, 2016), that correct responses were faster than incorrect responses. However, a coarser, but more inclusive temporal measure of delays (pauses before and during a response) was a better predictor of accuracy than response latency.

Out of the five effort cues examined in this study, four (hedges, delays, word fillers, and response latency) were significantly related to memory accuracy, but non-word fillers was not. Thus, our results largely mirror our hypotheses, as well as the results obtained by Lindholm et al. (2018). These results pointed in the same direction for all the cues, as correct statements contained fewer cues to retrieval effort compared to incorrect statements (see Figure 1). Furthermore, in the current study, hedges and delays proved to be unique predictors of accuracy. These results also concur with those of Lindholm et al. (2018), in that both delays and hedges uniquely predicted accuracy.

Previous research has demonstrated that response latency is reliably related to memory accuracy (Brewer et al., 2006; Koriat and Ackerman, 2010; Ackerman and Koriat, 2011; Weidemann and Kahana, 2016), and in the current study (in line with previous findings), correct responses were initiated faster than incorrect ones. However, including latency in the model did not make other effort cues redundant in predicting memory. Moreover, when comparing a model including response latency with a model including the coarser, but more inclusive measure of delays, the latter was found to explain more variance in accuracy than exact response latency. A plausible interpretation of this finding is that when memory retrieval unfolds as the memory is reported (Clark and Tree, 2002; Warren, 2012), delays during the response carry further information of retrieval effort and memory accuracy than that captured by the initial response latency. This result clearly calls for a reconsideration and broadening of how the temporal aspect of memory retrieval should be measured in future studies on cues related to memory accuracy.

As noted in the introduction, research suggests that people generally find it difficult to judge the accuracy of others’ memories (Lindholm, 2005, 2008a,b). An obvious practical question following from our findings is therefore whether practitioners, police officers and jurors in legal investigations, could be trained to use effort cues to better discriminate between honest witnesses’ accurate and inaccurate memories. While assessing memory accuracy based on signs of retrieval effort in an ongoing interview might prove difficult, the cues found to predict memory in our study should be fairly easy to learn to use when assessing accuracy from transcribed testimonies. Hence, a first step to test the practical value of the current findings would be to give evaluators instructions on cues related to accuracy, and then examine their performance in using these cues when assessing the accuracy of transcribed testimonies. While previous attempts modestly support the idea that instructions may improve accuracy of judgments (Koriat and Ackerman, 2010), research on the benefits of such training is scarce.

In the study by Lindholm et al. (2018), confidence did not contribute uniquely to variation in memory accuracy when controlling for effort cues. While we expected to replicate this finding, our study showed that confidence does indeed predict accuracy and also when effort cues were controlled for. Moreover, while the previous study demonstrated that effort cues fully mediated the relationship between accuracy and confidence, our results suggest partial mediation. Thus, although confidence in a memory may be partly based on cues to retrieval effort, our results suggest that there are other sources on which people base their confidence. In line with research findings within the framework of cue-utilization theory, candidates for these sources are likely found in the theory-based realm of cues, that is, in people’s beliefs and knowledge about memory (e.g., Matvey et al., 2001; Nussinson and Koriat, 2008). Moreover, it is reasonable to assume that retrieval effort is evident not only in the verbal and paraverbal cues studied here, but also in body language and facial mimicry (e.g., Krahmer and Swerts, 2005). Future studies should further scrutinize and include these potential alternative bases of confidence judgments and accuracy cues.

Despite replicating the main findings of Lindholm et al. (2018), there were also some differences between these studies. First, there is a slight variation between the studies regarding which specific cues contributed uniquely in predicting accuracy. For example, whereas non-word fillers in the Lindholm et al. (2018) study predicted accuracy, this cue was not significantly related to accuracy in our study. A straightforward explanation for this discrepancy is that effort cues vary in how reliably they are associated with memory. However, it could also be that the pattern of associations between cues and accuracy would become more stable with larger sample sizes.

### Limitations

While the interviews in our study were designed to simulate real eyewitness interviews, there are important limitations that restrict the generalizability of the findings to real world settings. First, we interviewed witnesses directly after they had viewed the crime event, meaning that the retention interval was negligible in comparisons to typical retentions between witnessing and reporting a target event in real-life eyewitness situations. Previous studies have demonstrated that factors that affect the discriminability of correct and incorrect memories, such as retention interval, may also change the relationship between response latency and accuracy (Brewer et al., 2006). Hence, an important issue for future studies is to examine how factors that affect discriminability (e.g., retention interval, task difficulty) may influence the validity of retrieval effort cues. Moreover, although our use of multi-level statistical analyses optimize power by taking advantage of the variability within individual witness responses, our sample of witnesses was admittedly small. Hence, our findings should ideally be replicated with larger samples. At the same time, the fact that research on semantic memory show effort/accuracy/confidence relationships with similar markers of effort (Smith and Clark, 1993) provides strong support for the validity of the current findings.

An important feature of this study was that measures of experienced effort were obtained during a natural, free-recall situation similar to that typical of eyewitness interviews. This meant that we asked them for confidence only after their recall of the whole event. While our procedure allowed witnesses to search their memory without being interrupted, this method may have had implications for their confidence ratings. For example, Robinson and Johnson (1996) showed that the confidence-accuracy relationship is stronger when estimating confidence after recalling an entire event, compared to immediately after each detail. Given that we replicate earlier findings of a positive confidence-accuracy relationship, it seems reasonable that our methodology did not bias the findings in any critical way. However, future studies should examine how procedural variations may affect the relations between confidence, accuracy, and effort cues.

Further, because the interviewer wrote down details reported by the witness during the ongoing interview, it was not possible for the interviewer to catch every single detail. This meant that confidence judgments could not be obtained for all statements. As we wanted to examine both effort cues and confidence in relation to memory accuracy, we decided to utilize the data for which confidence was also obtained. Thus, our analyses were carried out on a smaller dataset, not containing all statements provided by the witnesses. However, since the ratio of correct and incorrect statements were roughly the same for memories overall, and for memories with confidence estimates, we assume that the sample with confidence ratings is representative of the statements overall. For the interested reader, we have added analyses with the full dataset, excluding confidence in Supplementary Table 2.

In addition, while the instructions for coding of the effort cues were thoroughly pre-tested to be clear and unambiguous, the relatively low inter-rater reliability for some of the cues suggests that these instructions could be improved.

Finally, in this study our analyses focused on responses in the cued recall phase, which restricts our findings to this type of retrieval setting. Assuming that free recall memory primarily includes details that witnesses remember well, and hence retrieve fairly easy, it seems possible that effort cues might be less useful for discriminating accurate vs. inaccurate statements in this type of retrieval settings. This is one issue of obvious relevance for future research.

## Conclusion

Taken together, this study lends new support to the notion that retrieval effort in eyewitness responses is central for discriminating accurate from inaccurate recall of event details. Moreover, our findings suggest that a coarser, but more inclusive measure of delays before and during a response explains more variance in accuracy than response latency.

We show that effort cues partly mediate the relationship between accuracy and confidence, supporting the hypothesis that aspects of confidence are based on implicit, inferential processes. These findings suggest promising new ways of improving judgments of eyewitness evidence.

## Data Availability

All datasets generated for this study are included in the manuscript and/or the Supplementary Files.

## Ethics Statement

The study was conducted in full in accordance with the ethical principles outlined on http://www.codex.vr.se/, and with the 1964 Helsinki declaration and its later amendments. The studies did not include factors that require ethical vetting according to Swedish legislation on research ethics, http://www.epn.se/en/start/regulations/.

## Author Contributions

TL initiated, designed, and conducted data collection for the study. PG analyzed the data and wrote the manuscript in collaboration with TL and FJ. All authors contributed to interpretation of analyses and approved the final version of the manuscript.

## Conflict of Interest Statement

The authors declare that the research was conducted in the absence of any commercial or financial relationships that could be construed as a potential conflict of interest.
