# Supplementary material for: Predicting Accuracy in Eyewitness Testimonies With Memory Retrieval Effort and Confidence
Source: Front Psychol. 2019 Mar 29;10:703. doi: 10.3389/fpsyg.2019.00703 (PMC6450142; doi:10.3389/fpsyg.2019.00703)
Supplement: Supplementary file 1 [file Table_1.docx]

| Supplementary Table 1. Effort cues correlation matrix. | | | | |
| --- | --- | --- | --- | --- |
| Effort cue | 1 | 2 | 3 | 4 |
| 1. Response latency | – |  |  |  |
| 2. Delays | .67** | – |  |  |
| 3. Non-Word Fillers | .01 | .12** | – |  |
| 4. Word Fillers | .02 | .08** | .25** | – |
| 5. Hedges | .10** | .22** | .08* | .12** |
| * p <.05, ** p < .01 |  |  |  |  |
