# Supplementary material for: Predicting Accuracy in Eyewitness Testimonies With Memory Retrieval Effort and Confidence
Source: Front Psychol. 2019 Mar 29;10:703. doi: 10.3389/fpsyg.2019.00703 (PMC6450142; doi:10.3389/fpsyg.2019.00703)
Supplement: Supplementary file 2 [file Table_2.docx]

| Supplementary Table 2. Parameter estimates for predictors in models of accuracy (1043 observations). | | | | | | | | |
| --- | --- | --- | --- | --- | --- | --- | --- | --- |
|  |  |  |  |  |  |  |  |  |
| Predictor | Model 1 | Model 2 | Model 3 | Model 4 | Model 5 | Model 6 | Model 7^†^ | Model 8 |
|  |  |  |  |  |  |  |  |  |
| **Fixed effects** |  |  |  |  |  |  |  |  |
|  |  |  |  |  |  |  |  |  |
| Intercept | 1.13 (0.07)*** | 1.34 (0.09)*** | 1.23 (0.81)*** | 1.22 (0.08)*** | 1.47 (0.10)*** | 1.35 (0.10)*** | 1.63 (0.11)*** | 1.69 (0.12)*** |
|  |  |  |  |  |  |  |  |  |
| Delays |  | -0.68 (0.14)*** |  |  |  |  | -0.53 (0.15)** | - |
|  |  |  |  |  |  |  |  |  |
| Word Fillers |  |  | -0.25 (0.09)** |  |  |  | -0.17 (0.10) | -0.18 (0.10) |
|  |  |  |  |  |  |  |  |  |
| Non-word Fillers |  |  |  | -0.20 (0.10)* |  |  | - | - |
|  |  |  |  |  |  |  |  |  |
| Hedges |  |  |  |  | -0.56 (0.09)*** |  | -0.48 (0.09)*** | -0.52 (0.09)*** |
|  |  |  |  |  |  |  |  |  |
| Response latency |  |  |  |  |  | -0.17 (0.05)*** | - | -0.15 (0.05**) |
|  |  |  |  |  |  |  |  |  |
|  |  |  |  |  |  |  |  |  |
| **Random parameters** |  |  |  |  |  |  |  |  |
|  |  |  |  |  |  |  |  |  |
| Level 2 intercept  variance (participant) | <0.001 (<0.001) | <0.001 (<0.001) | <0.001 (<0.001) | <0.001 (<0.001) | 0.04  (0.19) | <0.001 (<0.001) | 0.02 (0.14) | 0.03 (0.16) |
|  |  |  |  |  |  |  |  |  |
| **Model fit** |  |  |  |  |  |  |  |  |
|  |  |  |  |  |  |  |  |  |
| Model df | 2 | 3 | 3 | 3 | 3 | 3 | 5 | 5 |
|  |  |  |  |  |  |  |  |  |
| Test change in df |  | 1^a^ | 1^a^ | 1^a^ | 1^a^ | 1^a^ | 2^b^ | 2^b^ |
|  |  |  |  |  |  |  |  |  |
| AIC | 1159.7 | 1137.1 | 1155.2 | 1157.9 | 1120.6 | 1159.6 | 1108.6 | 1112.9 |
|  |  |  |  |  |  |  |  |  |
| BIC | 1169.6 | 1151.9 | 1170.0 | 1172.7 | 1120.6 | 1135.5 | 1133.4 | 1137.7 |
|  |  |  |  |  |  |  |  |  |
| Akaike weight | 7.16× 10^-12^ | 6.79 × 10^-11^ | 4.79× 10^-11^ | 2.05× 10^-11^ | .002 | 7.53 × 10^-12^ | .89 | .10 |
|  |  |  |  |  |  |  |  |  |
| -2 log likelihood | -557.85 | -565.54 | -574.57 | -575.94 | -557.32 | -571.78 | -549.31 | -551.46 |
|  |  |  |  |  |  |  |  |  |
|  |  |  |  |  |  |  |  |  |
| Standard errors for fixed effects and standard deviations for random effects are given in parentheses. df = degrees of freedom. AIC = Akaike Information Criterion. BIC = Bayesian Information Criterion. ^†^ = Best-fitting model. Superscripts indicate df for the comparison between the current model and ^a^ Model 1 (baseline model), ^b^ Model 2-6. Asterisks indicate unique predictors within the model, *p <.05, **p < .01, ***p < .001. | | | | | | | | |
